# The Effects of In-Phase Bilateral Upper-Limb Exercises on Corticospinal Plasticity in People with Progressive Multiple Sclerosis: A Pilot Randomized Single-Case Concurrent Multiple-Baseline Design

**DOI:** 10.3390/brainsci16080782

**Published:** 2026-07-24

**Authors:** Marinos Chatzikonstantinou, Kyriaki Michailidou, Marios Pantzaris, Gavriella Alexandrou, Charalambos C. Charalambous, Dimitris Sokratous

**Affiliations:** 1 Physiotherapy Unit, Neurology Clinics, The Cyprus Institute of Neurology and Genetics, 2371 Nicosia, Cyprus; 2 Biostatistics Department, The Cyprus Institute of Neurology and Genetics, 2371 Nicosia, Cyprus; 3 Neuroimmunology Department, The Cyprus Institute of Neurology and Genetics, 2371 Nicosia, Cyprus; 4 Neurophysiology Department, The Cyprus Institute of Neurology and Genetics, 2371 Nicosia, Cyprus; 5 Department of Neurology, School of Medicine, Duke University, Durham, NC 27710, USA; charalambos.charalambous@duke.edu; 6 Department of Life and Health Sciences, Frederick University, 3080 Limassol, Cyprus

**Keywords:** progressive multiple sclerosis, in-phase bilateral, exercise, corticospinal excitability

## Abstract

**Highlights:**

**What are the main findings?**

- A 12-week in-phase bilateral upper-limb exercise protocol was safe, feasible and well tolerated in people with progressive multiple sclerosis.
- The intervention was associated with statistically significant improvements in corticospinal excitability and selected clinical outcomes in three out of five participants.

**What are the implications of the main findings?**

- In-phase bilateral upper-limb exercises may represent a low-cost, non-invasive, rehabilitation strategy with potential to support neuroplastic and functional improvement in progressive multiple sclerosis.
- In-phase exercise programs can be easily incorporated into routine clinical practice and group-based rehabilitation for people with progressive multiple sclerosis.

**Abstract:**

**Background:** Corticospinal plasticity is impaired in MS. In-phase bilateral upper-limb exercises have been shown to improve corticospinal excitability in people with relapsing-remitting multiple sclerosis, but there is limited literature that supports the same conclusion for progressive multiple sclerosis. Therefore, our aim was to investigate whether a 12-week in-phase bilateral upper-limb exercise protocol could enhance corticospinal plasticity and improve clinical outcomes in people with progressive multiple sclerosis. **Methods:** Five participants (two females; age = 61 ± 12.04 years) with secondary or primary progressive multiple sclerosis were randomized and recruited in a single-case concurrent multiple-baseline design study. The exercise protocol lasted for 12 consecutive weeks (30–60 min/session × 3 sessions/week) and incorporated adapted sport-specific activities, functional training and proprioceptive neuromuscular facilitation exercises. To define the functional relationship between the intervention and the results, a visual analysis was conducted. A statistical analysis was performed when a potential sizeable effect was observed. **Results:** Statistically significant reductions in active motor threshold (primary outcome) were observed in two of the five participants for the left upper limb and in one of the five participants for the right upper limb. Statistically significant improvements were partially observed in muscle strength, pinch strength and cognitive processing speed. **Conclusions:** Based on the preliminary findings of the current study, in-phase bilateral upper-limb exercises may modulate corticospinal plasticity and improve clinical outcomes in people with progressive multiple sclerosis. However, larger studies are needed to reproduce these results and to establish the effects of in-phase bilateral exercise on both corticospinal plasticity and related clinical outcomes.

## 1. Introduction

Multiple sclerosis (MS) is a chronic inflammatory, demyelinating, and neurodegenerative disease of the central nervous system that includes relapsing-remitting MS (RRMS) and progressive forms, such as secondary progressive MS (SPMS) and primary progressive MS (PPMS) [1,2]. Progressive multiple sclerosis (PMS) is characterised by gradual and irreversible neurological decline driven by chronic demyelination, axonal degeneration, and limited remyelination [3,4]. Although disease-modifying therapies can reduce inflammatory activity, they have limited effects on the neurodegenerative mechanisms underlying disease progression [5,6]. Consequently, there is an increasing need for neurorehabilitation strategies capable of promoting functional recovery and neuroplastic adaptation in people with PMS (pwPMS) [7].

Motor impairment in PMS is largely associated with structural damage to the corticospinal tract (CST) [8,9], one of the major descending pathways responsible for voluntary movement in humans [10,11,12,13]. Despite this damage, residual CST fibres may retain partial functional integrity and undergo activity-dependent reorganisation [14]. Neuroplastic mechanisms, including synaptic strengthening, axonal sprouting, and cortical remapping, may therefore support partial functional recovery when appropriately stimulated [13]. Impaired CST integrity and altered neuroplasticity are considered major contributors to motor dysfunction in PMS, making corticospinal plasticity an important therapeutic target [10,11,12,13,14,15].

Corticospinal plasticity can be assessed using transcranial magnetic stimulation (TMS), which provides quantitative measures of corticospinal excitability, including motor threshold and motor-evoked potential (MEP) amplitude [16]. Among these measures, active motor threshold (aMT) is considered a sensitive marker of corticospinal excitability and neuroplastic adaptation [17,18,19]. Individuals with progressive MS frequently demonstrate elevated aMTs compared to those with relapsing-remitting MS, reflecting reduced corticospinal excitability, demyelination, and axonal loss [20]. A higher aMT has also been associated with greater disability and slower neural conduction in PMS [20].

Exercise training is a promising, effective non-pharmacological approach for modulating neural excitability and improving motor performance [21,22,23]. In MS, exercise interventions have been associated with improvements in balance, walking ability, endurance, and fatigue, alongside reductions in motor threshold and increases in MEP amplitude [20,24,25,26]. Importantly, evidence suggests that CST may retain neuroplastic potential even in advanced disease stages [25]. Bilateral movement paradigms may further enhance neuroplasticity by promoting symmetrical cortical activation, reducing interhemispheric inhibition, and reinforcing weakened corticospinal projections [27,28,29]. Although bilateral in-phase training has shown promise in neurological populations such as those experiencing stroke [30], its effects on corticospinal excitability in PMS remain largely unexplored.

Therefore, the present study aimed to investigate whether a 12-week in-phase bilateral upper-limb exercise protocol could influence corticospinal plasticity and clinical function in five pwPMS. Specifically, the study examined whether the intervention could reduce aMT and improve indices of corticospinal excitability using a single-case concurrent multiple-baseline design [31,32,33]. It was anticipated that the findings would provide preliminary proof-of-concept evidence supporting the integration of bilateral exercise paradigms into neurorehabilitation strategies for progressive MS.

## 2. Materials and Methods

### 2.1. Participants

**Figure 1:**
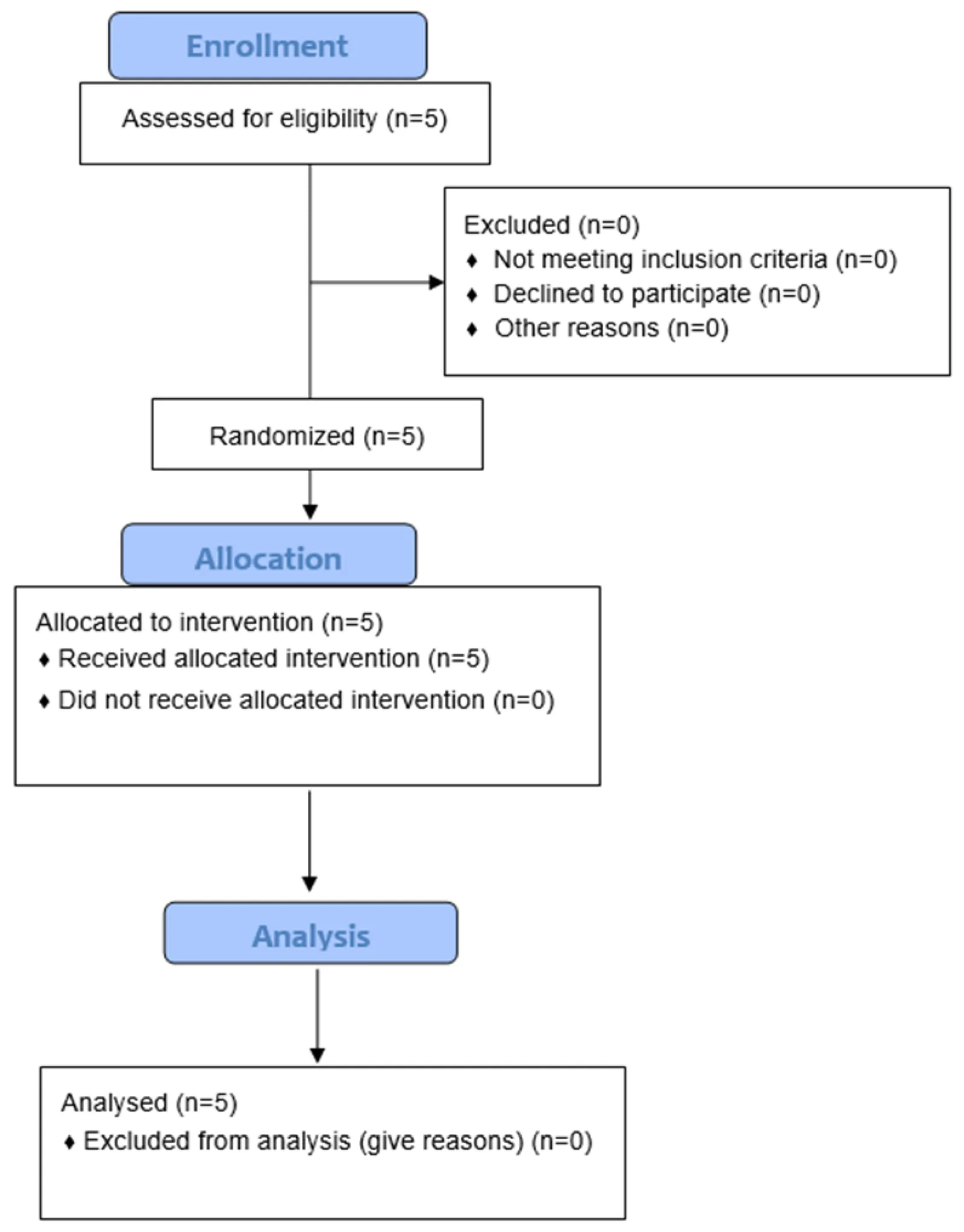
CONSOORT diagram: In line with the structure of a concurrent multiple-baseline design across subjects, the study involved five participants who met the pre-established inclusion criteria. All participants completed the full course of the intervention. No cases were excluded from the analysis. n: number of participants.

Participants were recruited and evaluated by a neurologist at the Cyprus Institute of Neurology and Genetics in February 2025 and were subsequently randomly enrolled (Figure 1). Individual medical records were collected on 1 March 2025; however, the authors did not have access to these records and were therefore unable to identify individual participants during or after data collection. The study was conducted from 10 March–13 August 2025.

The study protocol was approved by the Cyprus National Bioethics Committee (EEBK/ΕΠ/2022/32), and all procedures were conducted in accordance with its ethical guidelines. The study was registered at ClinicalTrials.gov (NCT07548242) prior to participant recruitment.

The inclusion criteria were: (1) diagnosis of PPMS or SPMS; (2) Expanded Disability Status Scale (EDSS) score between three and seven [34]; (3) age between 30 and 75 years. The exclusion criteria included (1) history of any other disease affecting the Central Nervous System other than PMS (e.g., Parkinson’s disease, stroke), (2) brain metal implants (e.g., aneurysm clips) [35], (3) history of cardiovascular diseases (e.g., heart failure, known aneurysm), (4) mental disorders (e.g., schizophrenia), (5) pregnancy during the implementation of the study, (6) history of epilepsy or seizures, (7) severe orthopaedic disorders (e.g., spine surgery, disk herniation, recent bone fractures), (8) visual deficit (e.g., optic neuritis, diplopia), (9) hearing impairments (e.g., deafness), and (10) spasticity level on upper or lower limbs more than 1+ (slight increase in muscle tone) according to the Modified Ashworth Scale [36].

All participants continued receiving their usual care, and they were instructed to continue their usual prescribed medications and maintain their regular daily routines throughout the study. They were also asked to refrain from engaging in any additional exercise protocol during the intervention period. Moreover, all participants read and signed a written informed consent form.

### 2.2. Study Design

**Figure 2:**
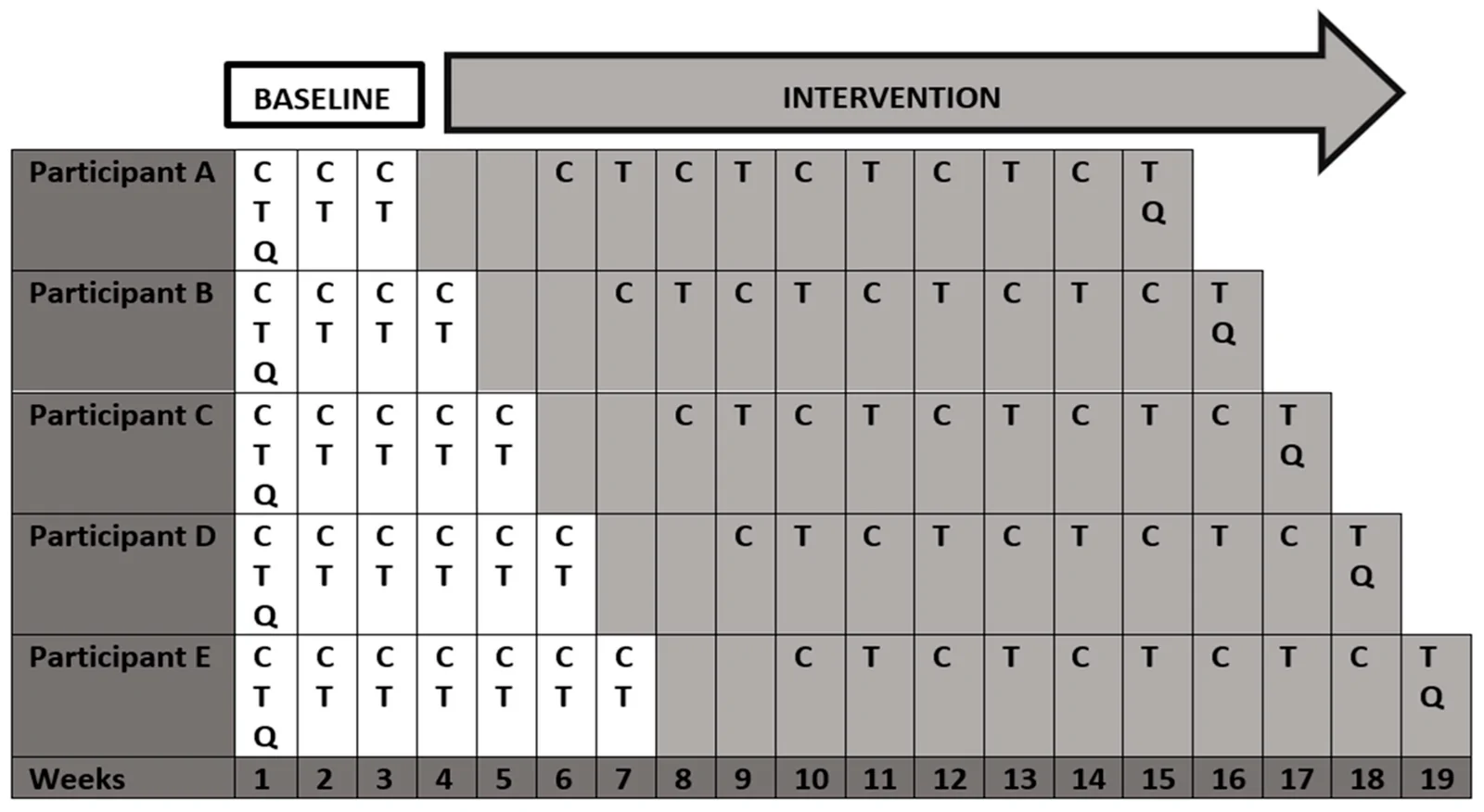
Study timeline: Each row represents a participant, and each column represents the corresponding week. The white-shaded weeks represent the baseline phase of the study, while the lighter grey-shaded weeks represent the intervention phase of the study. The design implements a staggered entry to control for time effects and ensure within-subject comparison across phases. C: clinical assessments; T: TMS assessments; Q: questionnaire administration (Modified Fatigue Impact Scale and Medical Outcome Study Questionnaire Short Form 36 Health Survey).

This study employed a concurrent multiple-baseline design across five pwPMS, without blinding, and was conducted in accordance with “single-case design” criteria [31,32,33]. According to established criteria, a minimum of three participants is required, with each providing at least three data points per variable of interest across the different study phases [31,33]. To enhance robustness in the presence of potential dropouts, five participants were enrolled. Multiple measurements were collected across phases: one assessment for each week during baseline and five during the intervention. All participants entered the baseline phase simultaneously, after which the intervention was introduced in a staggered manner across individuals and time (Figure 2). As each participant began the intervention, the others continued in baseline without treatment. This staggered implementation strengthens causal inference, as changes in outcomes occurred only after the introduction of the intervention, while measures remained stable in those still in the baseline phase. Consequently, the temporal pattern of the observed improvements is consistent with an effect of the intervention (i.e., in-phase bilateral upper-limb exercises), although alternative explanations cannot be completely excluded. To minimize the potential impact of fatigue on performance, all assessments and intervention sessions were carried out between 8:00 a.m. and 12:00 p.m. and followed the same order for all participants.

### 2.3. Baseline

The baseline phase started at the same time for all participants, and during that period, none of them underwent any other type of intervention. During the baseline phase, each participant completed the Modified Fatigue Impact Scale and the Medical Outcome Study Questionnaire Short Form 36 Health Survey during the first week. TMS measurement (aMT), as well as clinical assessments (see Secondary Outcome Measures), were then repeated on a weekly basis for all participants throughout the baseline period. The baseline phase lasted three weeks for the first participant, with each subsequent participant undergoing a progressively longer baseline period, increasing by a week (e.g., Participant A baseline period: 3 weeks; Participant B baseline period: 4 weeks, etc.).

### 2.4. Intervention

The intervention phase commenced immediately after completion of the last baseline week for each participant. The exercise protocol was based on the study by Sokratous et al. (2024) [24], with modifications to accommodate participants’ physical limitations. The program incorporated in-phase bilateral upper-limb movements, adapted sport-specific activities (e.g., basketball chest and overhead passes), functional exercises (e.g., squats, resisted seated hip abduction and adduction), and exercises based on the proprioceptive neuromuscular facilitation technique (Table 1) [37]. The training was delivered in a circuit format, consistent with established exercise guidelines for people with MS [7]. All sessions were performed under standardized safety measures, such as the use of mats, to reduce the risk of falls, and took place in a temperature-controlled setting (24 °C).

Each session consisted of three sets of nine upper-limb exercises targeting major muscle groups, including the shoulder flexors, extensors, abductors, adductors, and forearm flexors and extensors. Although the primary focus was on the upper limbs, three lower-limb exercises were interspersed after every three upper-limb exercises to allow for active recovery and to minimize upper-limb fatigue. A minimum of 20 repetitions was performed for each exercise. The total target volume for all upper-limb exercises was at least 720 repetitions per session, exceeding the 300–400 repetition threshold considered necessary to induce neuroplastic changes [38].

Each session lasted between 30 and 60 min and began with a five-minute warm-up and concluded with a five-minute cool-down to ensure safety and facilitate recovery. To maintain engagement and functional relevance, exercise difficulty (e.g., resistance of elastic bands, passing distance and number of repetitions) was periodically adjusted throughout the intervention. Heart rate was monitored at the end of each cycle to ensure that exercise intensity remained below 75% of the age-predicted maximum heart rate, thereby avoiding aerobic training zones [39].

The intervention was delivered over 12 consecutive weeks, comprising a total of 36 sessions. To ensure adequate exposure, participants were required to attend at least 75% of the scheduled sessions (minimum 27 out of 36) [40]. Participants who did not meet this attendance criterion were excluded from the final analysis to preserve the reliability and interpretability of the outcome measures.

### 2.5. Primary Outcome Measure

Given that the aMT is a valid and important index of corticospinal plasticity in pwPMS [16,17,18,19] and considering that the primary aim of this study is to determine whether a 12-week in-phase bilateral upper-limb exercise program modulates aMT, this measure was selected as the primary outcome to reflect changes in corticospinal plasticity. Lower aMT measures compared with baseline measures represent improved corticospinal excitability [18,41]. Based on our previous research [24], the only measure that showed statistically significant changes in MS participants was the motor threshold; thus, it was used as the primary outcome measure in this study.

### 2.6. Secondary Outcome Measure

The secondary outcome measures included several clinical assessments, such as the Timed 25-Foot Walk Test (test measuring gait speed), in which lower measures indicate improvement in walking speed; the Mini-Balance Evaluation System Test (evaluates dynamic balance), in which higher measures indicate improvement in balance; the Purdue Pegboard Test (assessment of upper-limb dexterity), in which higher measures indicate improved manual dexterity; the Hand-Held Dynamometer and Pinch Test (assesses muscle strength), in which higher measures indicate improved muscle strength; the Symbol Digit Modalities Test (cognitive processing speed assessment), in which higher measures indicate improved cognitive processing speed; the Modified Fatigue Impact Scale (defines fatigue level), in which lower measures indicate reduced perceived impact of fatigue; and Medical Outcome Study Questionnaire Short Form 36 Health Survey (assesses health-related quality of life), in which higher measures indicate improvements in self-reported health-related quality of life. According to “single-case design” guidelines, two physiotherapists independently performed all clinical assessments on each participant.

### 2.7. Data Acquisition of Outcome Measures

#### 2.7.1. TMS Outcome Measures

Corticospinal plasticity was assessed using single-pulse TMS in the neurophysiology laboratory of the Cyprus Institute of Neurology and Genetics. MEPs were recorded via surface EMG from the abductor pollicis brevis (APB). Single-pulse TMS was used to elicit MEPs, which were subsequently used to calculate the aMT. The TMS procedures, described in detail below, were identical for all participants across all assessment time points and were applied bilaterally to the APB.

Participant positioning: During all TMS assessments, participants were seated comfortably in a relaxed position, with both arms supported on cushioned armrests, their feet flat on the floor, and their head resting against a cushion to maximize comfort and minimize unwanted movement. All neurophysiological assessments were conducted under these standardized conditions. To ensure methodological consistency, data were collected using identical procedures for all participants and across all time points.

EMG: To record EMG activity from the APB, standardized procedures [18] for participant preparation and data acquisition were followed. Prior to electrode placement, the skin over the palm was cleaned with alcohol pads to reduce impedance. Surface electrodes were then positioned over the APB, with the active (anode) electrode placed over the distal portion of the muscle belly and the reference (cathode) electrode positioned proximally along the median nerve branch innervating the APB. A ground electrode was placed over the lateral humeral condyle of the tested upper limb [18,41]. EMG signals were acquired using the KeyPoint Net Electromyography system (version 2.40; Natus Medical Incorporated G4, Middleton, WI, USA).

TMS assessment: Stimulation was performed using a figure-of-eight coil (C-B60; 35 mm inner diameter, 75 mm outer diameter) connected to a MagPro R20 magnetic stimulator (MagVenture A/S, Farum, Denmark, UK edition). The coil was positioned tangentially over the primary motor cortex, contralateral to the targeted APB. The handle was oriented posterolaterally at approximately 45° to the midline to produce a posterior–anterior induced current [18,42,43].

For the TMS procedures, the “hotspot” was first identified [18], followed by the determination of aMT. Once the hotspot was identified, it was marked on the scalp using a water-resistant marker to ensure consistent coil placement during the session. These individual anatomical measurements were repeated before each TMS assessment to maintain accuracy.

Hotspot Localisation: The motor hotspot was defined as the cortical site eliciting the largest MEP in the APB. It was identified by single-pulse TMS, beginning at a low stimulation intensity (~45% of the maximum stimulator output) and increasing in increments of 1–5% [17]. Stimulation intensity was progressively increased until three consecutive MEPs with peak-to-peak amplitudes matching or exceeding 200 µV [41] were achieved during voluntary muscle contraction. Muscle pre-activation was maintained at 15% of the APB’s one-repetition maximum [41], which was established beforehand using a standardized pinch force test (JAMAR plus+ Sammons Preston, Bolingbrook, IL, USA).

Active Motor Threshold Detection: Following hotspot localization, bilateral aMTs were assessed. The aMT was estimated using the Motor Threshold Assessment Tool 2.0 (MTAT 2.0) [18], which employs an adaptive threshold-hunting algorithm. A total of 15 single-pulse stimulations [18,42] were delivered, with the APB pre-activated using the pinch test in the same manner as during hotspot identification for all participants. The minimum intensity required to elicit MEPs with a minimum peak-to-peak amplitude of 200 µV was considered the aMT [18,42].

#### 2.7.2. Clinical Assessments

1. **Timed 25-Foot Walk Test**

This test is used as a standardized performance-based measure of ambulatory function [44]. Participants were instructed to safely walk a clearly marked 25-foot path as quickly as possible. The test was performed twice, and the average time was recorded as the final score. A minimum change of 20% between pre- and post-intervention measurements was considered statistically and clinically meaningful [45].

2. **Mini-Balance Evaluation System Test**

This is a 14-item clinical tool designed to evaluate dynamic balance across four domains: anticipatory postural adjustment, reactive postural control, sensory orientation and dynamic gait [46]. Each item is scored on a 3-point scale (0, 1, 2) with the highest scores indicating better balance. This test has demonstrated strong convergent validity, and it has also shown good sensitivity in distinguishing fallers from non-fallers among people with MS [47].

3. **Purdue Pegboard Test**

Manual dexterity is assessed using the Purdue Pegboard Test. All participants were tested individually in a quiet room, seated comfortably at a table, with the pegboard positioned directly in front of them at midline. Standardized instructions were provided prior to each subtest, and a practice demonstration was given when necessary to ensure task comprehension. The test consists of four subtests administered in the following order: (1) the unilateral dominant-hand test, in which participants use only their dominant hand to place as many pins as possible into the holes of the board within 30 s. Pins are taken one at a time from the corresponding cup and inserted sequentially from top to bottom, and the total number of correctly placed pins is recorded; (2) the unilateral non-dominant-hand test, which follows the same procedure as the dominant-hand test, except that participants use only their non-dominant hand; (3) the bimanual test, in which participants use both hands simultaneously to place pins into the two columns within 30 s, and the total number of correctly placed pin pairs is recorded; and (4) the assembly test, which assesses bilateral fine motor coordination, sequencing ability, and higher-order cognitive control, during which participants use both hands to assemble a sequence consisting of a pin, washer, collar, and washer in the correct order within 60 s, and the total number of correctly completed assemblies is recorded. This test has high reproducibility without learning effects and correlates with disability levels and response to upper-limb rehabilitation in people with MS [48].

4. **Hand-Held Dynamometer**

To assess the muscle strength of major upper-limb muscle groups, a hand-held dynamometer (Kinvent Biomechanique, Montpellier, France), which provides real-time biofeedback, was used [49]. Participants were positioned in the supine position and were asked to perform maximal isometric contractions of specific upper-limb muscle groups (i.e., shoulder flexors and extensors, internal and external rotators, adductors and abductors, horizontal adductors and horizontal abductors, and elbow flexors and extensors). The KFORCE App (Kinvent Biomechanique, Montpellier, France) was used to record and store individual data.

5. **Symbol Digit Modalities Test**

This is a widely used test in people with MS, assessing information-processing speed [50]. Participants were asked to match symbols with numbers and try to complete as many correct pairings as possible within 90 s. The written form of the test was used. To minimize the risk of participants becoming familiar with the symbol-digit associations, different versions of the written form of the SDMT were used across sessions [51]. This test was performed every week of the baseline phase and used as a clinical assessment test during intervention phase assessments. This was done in order to enhance measurement stability and reduce the influence of transient factors (e.g., fatigue, symptom variability). This test has demonstrated strong contrast validity in people with MS, with moderate correlations to physical disability measures, such as those noted for the EDSS [52].

6. **Pinch Test**

The Jamar digital pinch gauge (Sammons Preston, Bolingbrook, IL, USA) was used to assess pinch strength. During the test, participants were instructed to pinch the device with maximal effort. To minimize measurement error, participants’ fingers were positioned in the middle of the measurement surface, and three trials were performed [53]. This device is a valid measure of fine motor function and strength, demonstrating high test–retest reliability (ICC > 0.90), supporting its applicability in conditions involving upper-limb motor deficits [53].

7. **Modified Fatigue Impact Scale**

This is a subjective questionnaire which evaluates the impact of fatigue on physical, cognitive and psychological functioning [49]. It has shown strong convergent validity with the Fatigue Severity Scale and is particularly sensitive to the physical domain of fatigue, which overall, correlates with fatigue severity. The higher the score, the more significant the negative effect from fatigue in the person’s everyday life [54].

8. **Medical Outcome Study Questionnaire Short Form 36 Health Survey**

This is a subjective generic questionnaire evaluating eight domains of physical and mental health, including limitations in physical functioning restrictions in role activities due to physical health, restrictions in role activities due to emotional problems, general mental health bodily pain limitations in social functioning, fatigue, and overall health perceptions [55]. It consists of 11 subjectively rated questions. Scores range from 0 to 4, with lower values indicating greater disability and higher scores reflecting better functioning and less disability.

### 2.8. Data Analysis

To investigate the effects of the proposed exercise protocol, recommended guidelines were followed [31,32,33]. Separate analyses were conducted for each outcome measure across all experimental phases (i.e., baseline and intervention). First, a visual analysis was performed to determine whether a functional relationship existed between the intervention and the outcome measures. Subsequently, statistical analyses were conducted for all outcomes that demonstrated a substantial effect in the visual analysis to evaluate the magnitude of the intervention effect.

### 2.9. Visual Analysis

Two independent evaluators recorded all clinical outcome measures over time, and inter-rater reliability was assessed for a minimum of 20% of the data points in each condition. An inter-rater agreement of at least 0.80 was considered acceptable [33]. To evaluate the effectiveness of the proposed exercise protocol, visual analysis was conducted, following established single-case experimental design guidelines [31,32]. Initially, a visual analysis was performed, and the data were presented graphically using spaghetti plots to determine whether a functional relationship existed between the intervention and the primary outcome measure [31,32,33].

During the visual analysis, seven features of the graphed data were examined: level, trend, stability, variability, immediacy of effect, overlap and consistency [31,32,33]. Level refers to the average value of the outcome measure within each phase and indicates the overall magnitude of performance. To quantify the between-phases changes in level and thus, to identify whether there is considerable increase in the targeted outcomes, the Percentage of Data Exceeding the Median (PEM) method was used [56]. The PEM quantifies the proportion of intervention-phase data points that surpass the baseline median in the expected direction of change. A PEM value of ≥80% is generally considered indicative of a strong, consistent intervention effect. Trend captures the direction of data change over time (e.g., increasing, decreasing or stable) within a phase. Stability reflects the degree of variability in the data, with stable phases containing most of the data points within ±15% of the median. Variability refers to the degree of fluctuation or spread of data points within a phase around the median, with ≤20% as an acceptable measurement. The immediacy of effect was evaluated by observing how quickly the behaviour changes following the introduction or withdrawal of the intervention. Overlap examines the extent to which data points from adjacent phases (i.e., baseline and intervention) are shared. Lower overlap suggests a stronger intervention effect. Finally, consistency refers to the replication patterns (e.g., level shifts or trends) across different participants supporting the generalizability of findings [33].

### 2.10. Statistical Analysis

Visual analysis was conducted for each outcome variable to assess potential effects of the proposed exercise protocol. Consistent with current single-case experimental design recommendations [31,32,33], visual analysis served as the primary analytical approach and was complemented by quantitative analyses, including the non-overlap of all pairs (NAP) effect size and randomization tests, to strengthen the objectivity of the findings. When visual inspection suggested possible functional changes between the baseline and intervention phases, based on the seven key features described in the visual analysis framework, the NAP metric was used to estimate effect size. For outcomes in which lower scores represented improvement, such as aMT, NAP values approaching 0% were interpreted as complete non-overlap in the expected direction, indicating that intervention values were consistently lower than baseline values. Randomization tests were also performed to evaluate statistical significance [31,32,33]. The null hypothesis stated that the intervention would have no effect, implying that participants’ responses were independent of the condition (baseline versus intervention). The alternative hypothesis proposed that the neurophysiological and/or clinical parameters would be influenced by the intervention. The null hypothesis was rejected if the *p*-value was below the Bonferroni-adjusted significance threshold (0.05 divided by the number of tests conducted). All tests were two-sided. Statistical analyses were performed using R 4.6.1. (https://www.r-project.org).

## 3. Results

All five participants successfully completed the 12-week exercise protocol, three sessions per week, for an average of 45 min per session across subjects, without any side effects and no missing assessments. The exercise protocol was well tolerated, and no adverse events were reported. Given that there were no dropouts and no missing assessments, the full dataset was included in the final analysis. The effects of the exercise protocol on the aMT and clinical outcomes were initially examined through visual inspection of individual performance trajectories. When visual analysis suggested potential functional effects and met the established criteria (i.e., level, trend, stability, variability, immediacy of effect, overlap, and consistency) between the baseline and intervention phases, the NAP metric was subsequently applied to quantify intervention effects. All outcomes were analysed bilaterally to capture potential neuroplastic and functional changes induced by the proposed exercise protocol.

All data can be found in the FIGSHARE repository (https://doi.org/10.6084/m9.figshare.32098522, accessed on 25 April 2025). Demographics and baseline clinical characteristics of the participants were collected prior to the intervention and are presented in Table 2.

### 3.1. Results of Visual Analysis

#### 3.1.1. Primary Outcome Measure—Active Motor Threshold

A decrease in aMT signifies an improvement in corticospinal plasticity. The results of the aMT assessments during all time points are presented in Appendix A.

#### 3.1.2. Within-Phase Analysis

**Figure 3:**
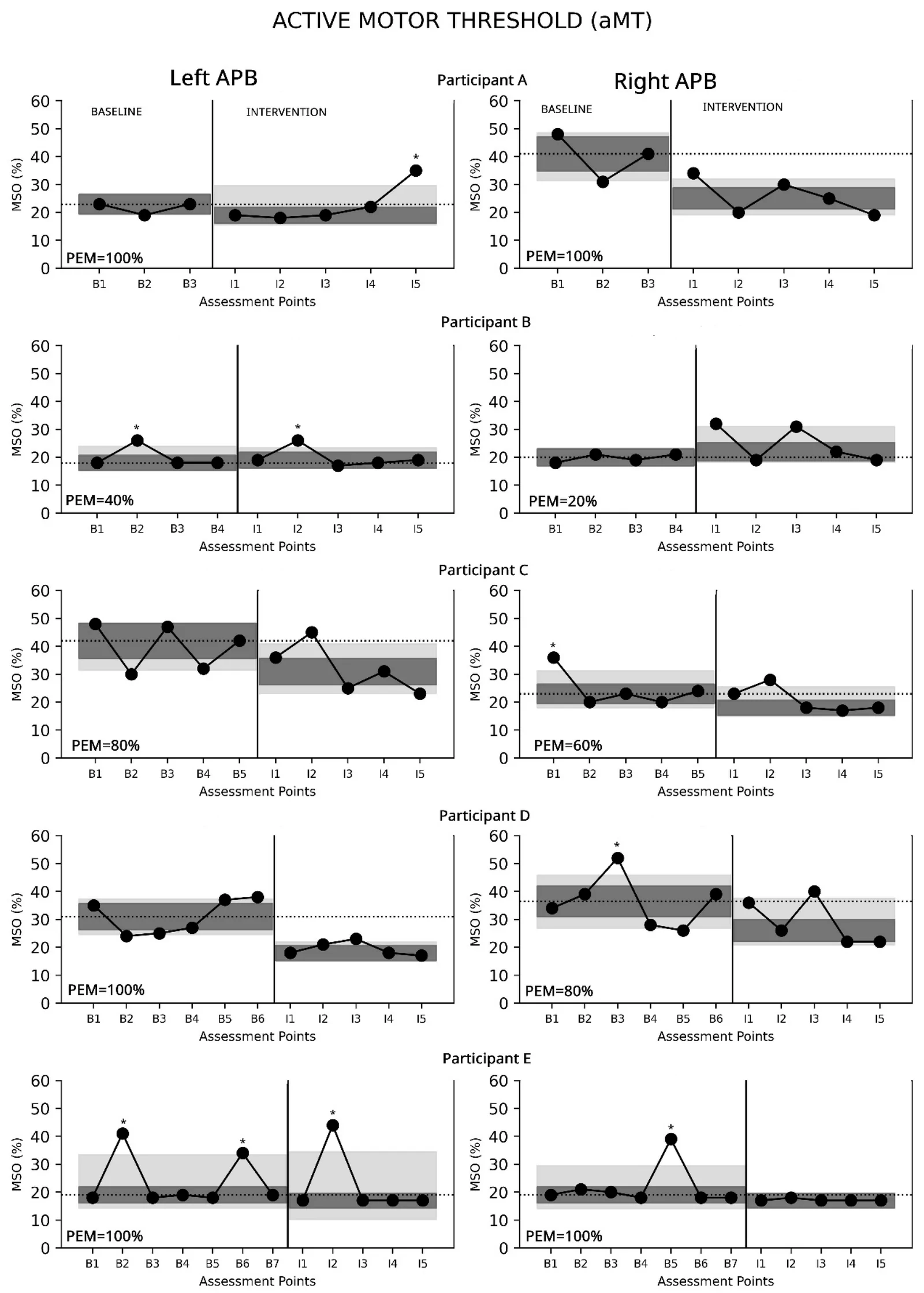
Active Motor Threshold: Graphs represent the left (left column) and right (right column) upper limb of each participant, as assessed through the abductor pollicis brevis (APB). The x-axis presents the assessment points for the baseline (B) and intervention (I) phases, while the y-axis represents the MSO (%). The darkest grey shaded areas indicate the stability ranges, and the lighter grey shaded areas indicate the variability ranges. In cases where only one shaded area is visible, this is due to overlap between stability and variability ranges. (*) in the graphs indicate outliers that were excluded from the analysis. The dotted lines represent the medians of each phase, with the baseline median extending across both phases, as it is used for the calculation of PEM. The vertical black line separates the baseline phase (**left**) from the intervention phase (**right**). APB: abductor pollicis brevis; B: baseline; I: intervention; PEM: percentage exceeding the median; MSO: maximum stimulator output.

Data from both the left and right upper limbs were analysed in terms of variability, stability, and trend, as well as the median of baseline phase (Figure 3). Data variability was classified as low (≤20% of points outside the stability range), moderate (20–40%), or high (>40%) [31,32,33]. Low variability was expected bilaterally in both the baseline and intervention phases. Stability of the data was defined as having more than 80% of data points within ±15% of the phase median [31,32,33]. Trend was assessed based on the direction of change in data across each phase [31,32,33]. A stable trend was expected during the baseline phase, whereas during the intervention phase, a downward trend, or at least a stable trend accompanied by a level change, was anticipated. The median (M) was calculated to provide a reference point for comparing the percentage of PEM between the baseline and intervention phases.

### 3.2. Participant A

The fifth assessment point of the left APB during the intervention phase was identified as an outlier and was therefore excluded from the analysis (Figure 3).

#### 3.2.1. Left APB

The median during the baseline phase was calculated at M = 23, with low variability (17%) and a stable trend observed; however, stability (66.7%) was not achieved. In contrast, the median score during the intervention phase was calculated as M = 19, showing high variability (58%) and stability (75%), although an upward trend was observed.

#### 3.2.2. Right APB

During the baseline phase (M = 41), moderate variability (34.9%), low stability (33.3%) and a downward trend were observed. In the intervention phase (M = 25), high variability (44.9%) was observed, but with low stability (20%) and a downward trend.

### 3.3. Participant B

The second assessment point during the baseline phase and the second assessment point during the intervention phase of the left APB were identified as outliers and were therefore excluded from the analysis (Figure 3).

#### 3.3.1. Left APB

Both the baseline (M = 18) and intervention (M = 19) phases demonstrated low variability (0% and 11%, respectively) and high stability (100%). The baseline phase exhibited a stable trend, while the intervention phase showed a tendency toward a decreasing trend.

#### 3.3.2. Right APB

During the baseline (M = 20), variability was low (15%), with high stability (100%), but an upward trend was observed. In the intervention phase (M = 22), high variability (50%) and low stability (60%) were observed, while the trend shifted downward.

### 3.4. Participant C

The first assessment point of the right APB during baseline was considered an outlier and was thus excluded from the analysis (Figure 3).

#### 3.4.1. Left APB

During the baseline phase (M = 42), high variability (41%) and low stability (60%) were observed, with a slightly decreasing trend. In the intervention phase (M = 31), variability remained high (54.8%), and stability further decreased (20%), accompanied by a downward trend.

#### 3.4.2. Right APB

Stability was 60% in both phases (baseline, M = 21.5; intervention, M = 18), with moderate variability at baseline (35%) and high variability during intervention (54%). Both phases demonstrated a downward trend.

### 3.5. Participant D

The third assessment point of the right APB during baseline was considered an outlier and was thus excluded from the analysis (Figure 3).

#### 3.5.1. Left APB

Baseline data (M = 31) exhibited moderate variability (39%) and low stability (33.3%), with an overall upward trend. During the intervention phase (M = 18), variability decreased (25%), and stability increased (60%). Data collected during the intervention phase demonstrated a downward trend.

#### 3.5.2. Right APB

Stability was 60% in both the baseline (M = 34) and intervention (M = 26) phases. Variability was moderate during the baseline phase (35%) and increased to high levels during the intervention phase (54%). A downward trend was observed across both phases.

### 3.6. Participant E

The second and sixth assessment points of Participant E during the baseline phase, as well as the second assessment point during the intervention phase for the left APB and the fifth baseline assessment point for the right APB, were identified as outliers and were therefore excluded from the analysis (Figure 3).

#### 3.6.1. Left APB

Participant E’s left APB measurements demonstrated high stability (100%) and low variability (baseline = 6%, intervention = 0%) across both study phases (baseline, M = 18; intervention, M = 17). Both the baseline and intervention phases showed a downward trend.

#### 3.6.2. Right APB

Similar to the left APB, the data for the right APB were highly stable in both phases (100% stability) and exhibited low variability (baseline = 12.3%; intervention = 5.2%) (baseline, M = 18.5; intervention, M = 17). The baseline phase results demonstrated a slight upward trend, whereas the trend during the intervention phase remained stable.

### 3.7. Between-Phases Visual Analysis

The visual analysis was based on the following criteria: (1) a stable trend during baseline and a downward or stable (with change in level) trend during the intervention phase [31,32,33]; (2) baseline stability of at least 80% (i.e., ≥80% of data points within 15% of the phase median) [31,32,33]; (3) low baseline variability (≤20%) [31,32,33]; (4) a clearly identifiable immediacy of effect immediately after the phase transition [31,32,33]; (5) data overlap of ≤50%between the baseline and intervention phases [31,32,33]; (6) consistency of intervention phase data [31,32,33]; (7) a level change in the expected direction (i.e., decrease for the aMT); and (8) a PEM value of ≥80%, indicating that the majority of the intervention phase data points exceeded the baseline median in the expected direction (i.e., below the PEM line for the aMT) [31,32,33].

### 3.8. Participant A

#### 3.8.1. Left APB

Regarding the left APB, there was a level decrease (3.0%). At the beginning of the intervention phase, the first three data points showed an immediate downward shift; however, a late-phase spike disrupted the overall consistency. The data overlap was 60%, and the PEM was 100%.

#### 3.8.2. Right APB

A larger decrease in level (12.0%) was observed, accompanied by an immediate effect at the phase transition. The data remained consistently lower throughout the intervention, with a 40% overlap and a PEM of 100%.

### 3.9. Participant B

#### 3.9.1. Left APB

There was no level change across phases. Consistency declined from high to moderate, and overlap was 75%. PEM was calculated at 40%.

#### 3.9.2. Right APB

The intervention phase included a level increase (7.0%) and immediacy of effect. However, consistency decreased from moderate to low. The overlap was 40%, and PEM reached 40%.

### 3.10. Participant C

#### 3.10.1. Left APB

A reduction of level (5.0%) was observed, with a moderate immediacy of effect. The intervention data showed moderate consistency, with an overlap of 60%. PEM was 80%.

#### 3.10.2. Right APB

A slight increase in level (0.67%) was detected between phases. There was no immediacy of effect, and consistency was low, with an overlap of 60% and 60% PEM.

### 3.11. Participant D

#### 3.11.1. Left APB

A level decrease was observed, with a decrease of 13.3% and an immediate drop at the beginning of the intervention phase. The data remained consistently low, with no overlap between phases and with 100% PEM.

#### 3.11.2. Right APB

In contrast, the right APB showed an increased level (3.0%), with weak and inconsistent datapoint trajectories across the intervention phase. Moreover, a 40% overlap was observed, with 80% PEM.

### 3.12. Participant E

#### 3.12.1. Left APB

A reduction of level (1.67%) was observed, accompanied by an immediate drop at the beginning of the intervention. The data exhibited consistency throughout the intervention phase. There was no overlap between phases (0%), and PEM was 100%.

#### 3.12.2. Right APB

A reduction in level (0.67%) and an immediate drop in the intervention phase were observed. Intervention data also exhibited high consistency, with a 20% overlap between phases and with 100% PEM.

Based on the visual analysis criteria, three datasets from the cohort met the inclusion criteria [31,32,33] and were therefore included in the statistical analysis. These comprised the right APB of Participant A, the left APB of Participant D and the left APB of Participant E.

### 3.13. Secondary Outcome Measures—Clinical Assessments

#### 3.13.1. Timed 25-Foot Walk Test

##### Within-Phase Visual Analysis

Participant A showed 100% stability at baseline (M = 1.4), with 47.5% (high) variability and a stable trend, while in the intervention phase (M = 1.2), stability was 80%, variability 63.8% (high), and a downward trend was observed. Participant B demonstrated 50% stability at baseline (M = 11.3), with 40.1% (moderate) variability and a downward trend, while in the intervention phase (M = 10.5), 100% stability, 10.5% (low) variability and a downwards trend were observed. Participant C showed 100% stability in both phases (baseline, M = 20; intervention, M = 17.3), with 15.9% (low) variability and a stable trend at baseline, whereas during the intervention phase, the results showed 6.4% (low) variability and a stable trend. Participant D also showed 100% stability across phases (baseline, M = 15.2; intervention, M = 13.9), 20.3% (low) variability and stable trend, whereas during the intervention phase, variability was 24.2%, (low), with a downward trend. Participant E showed stability on both phases (baseline, 99.8%; intervention, 99.5%) (baseline, M = 7.2; intervention, M = 4.9), 16.2% (low) variability and an upward trend, while during the intervention, variability was 45.7% (high), with a downward trend.

##### Between-Phase Visual Analysis

Participant A showed a PEM of 40%, low immediacy of effect, and low consistency across data points. A slight decrease in level of 0.02 s was observed, accompanied by a 60% overlap between phases, suggesting minimal differentiation between the baseline and intervention phases. Participant B demonstrated a PEM of 100%, immediacy of effect between phases, moderate consistency, an increase of 0.27 s, and no overlap. Participant C showed a PEM of 100%, an immediate effect, moderate consistency, and a notable level decrease of 3.47 s, with no overlap between phases. Participant D also showed a PEM of 100%, a strong immediacy of effect, upward consistency, a positive level change of 0.47 s, and a 60% overlap between phases. Participant E demonstrated a PEM of 80%, small immediacy of effect, moderate consistency, a level shift of 0.29 s, and a 20% overlap between phases.

### 3.14. Mini-BEST Test

#### 3.14.1. Within Phase Visual Analysis

All participants showed 100% stability of the data for both phases. Participant A showed baseline (M = 11) variability at 18.7% (low) and an upward trend, while in the intervention phase (M = 11), variability was 1.9% (low) and showed a stable trend. Participant B showed a variability of 4.8% (low) at baseline (M = 21) and 9.4% (low) at intervention (M = 23). Both phases had a stable trend. Participant C showed a baseline M = 20 variability of 7.1% (low) with a stable trend, while intervention (M = 21) variability was 10.7% (low) with an upward trend. Participant D exhibited a baseline (M = 23) variability of 3.8% (low) with a stable trend and an intervention (M = 24) variability of 12.8% (low) and an upward trend. Participant E showed a baseline (M = 28) variability of 2.7% (low) with an upward trend and 0% (low) during intervention (M = 28).

#### 3.14.2. Between-Phase Visual Analysis

Participant A showed a PEM at 40%, no immediate effect, strong consistency during intervention, and a level of increase of 0.73 points with 60% overlap. Participant B demonstrated a PEM at 100%; an immediate effect was observed, as well as strong consistency across phases, with a level increase of 2 points with no overlap. Participant C displayed a PEM of 80%, an immediate effect, low consistency, and a modest level increase of 0.67 points with 20% overlap. Participant D demonstrated a PEM at 80%, immediate effect, strong consistency, and a level increase of 1.7 points with 40% overlap. Participant E showed no changes, with a PEM at 0%, no immediate or consistent changes, no level shift, and 100% overlap.

### 3.15. Purdue Pegboard Test

#### 3.15.1. Within-Phase Visual Analysis

Participant A showed 33.3% stability, variability of 54.4% (high), and a decreasing trend during the baseline phase (M = 3) of the dominant hand subtest. Regarding the non-dominant hand subtest (M = 3), the stability was 66.7%, and variability was 17.7% (low), with an increasing trend. Regarding the bimanual subtest (M = 1), the stability was also 66.7%, with variability at 70.7% (high) and an increasing trend. Lastly, regarding the interchange subtest (M = 7), the stability was 100%, with variability at 11.7% (low) and an increasing trend. During the intervention phase, the dominant hand subtest (M = 6) showed 60% stability, with variability at 8.7% (low) and an increasing trend. Regarding the non-dominant hand (M = 2), the stability was 20%, and variability was at 31.6% (moderate), with an increasing trend. Regarding the bimanual subtest (M = 1), the stability was 60%, with no variability at 0% and a flat trend. Regarding the interchange subtest (M = 11), the stability was 60%, variability was 18.2% (low), and the trend was increasing.

Participant B showed 75.0% stability, 11.8% (low), variability, and a decreasing trend during the baseline phase (M = 12.5) of the dominant hand subtest. Regarding the non-dominant hand subtest (M = 12), the stability was 100 and variability was 4.3% (low), with a flat trend. Regarding the bimanual subtest (M = 8.5), the stability was 50%, with variability at 15.2% (moderate) and a decreasing trend. Lastly, regarding the interchange subtest (M = 18), the stability was 100%, with variability at 8.2% (low) and a decreasing trend. During the intervention phase, the dominant hand subtest (M = 12) showed 80% stability, with variability at 7.2% (low) and a flat trend. Regarding the non-dominant hand (M = 12), the stability was 100% and variability was at 6.9% (low), with an increasing trend. For the bimanual subtest (M = 10), the stability was 100%, with variability at 5.7% (low) and an increasing trend. Regarding the interchange subtest (M = 19), the stability was 80%, variability was at 13.2% (low), and the trend was increasing.

Participant C showed 60% stability, 37.3% (moderate) variability, and an increasing trend during the baseline phase (M = 4) of the dominant hand subtest. Regarding the non-dominant hand subtest (M = 3), the stability was 40.0% and variability was at 39.5% (moderate), with a decreasing trend. Regarding the bimanual subtest (M = 2), the stability was 80.0%, with variability at 28.3% (moderate) and an increasing trend. Lastly, regarding the interchange subtest (M = 3), the stability was 60.0%, with variability at 54.7% (high) and an increasing trend. During the intervention phase, the dominant hand subtest (M = 4) showed 60% stability with 25.0% (moderate) variability and a decreasing trend. Regarding the non-dominant hand (M = 3), the stability was 40% and variability was at 42.2% (moderate), with an increasing trend. Regarding the bimanual subtest (M = 2), the stability was 40%, with variability at 33.3% (moderate) and a decreasing trend. Regarding the interchange subtest (M = 6), the stability was 80%, variability was at 6.3% (low), and the trend was flat.

Participant D showed 66.7% stability, 15.9% (moderate) variability, and an increasing trend during the baseline phase (M = 6.5) of the dominant hand subtest. Regarding the non-dominant hand subtest (M = 7), the stability was 83.3% and variability was at 12.7% (low), with a slight increasing trend. Regarding the bimanual subtest (M = 4.5), the stability was 66.7%, with variability at 20% (low) and a slight increasing trend. Lastly, regarding the interchange subtest (M = 13.5), the stability was 66.7%, with variability at 17.3% (low) and a slight decreasing trend. During the intervention phase, the dominant hand subtest (M = 8) showed 100% stability, with variability at 10.3% (low) and an increasing trend. Regarding the non-dominant hand (M = 8), the stability was 80% and variability was at 10% (low), with an increasing trend. For the bimanual subtest (M = 5), the stability was 80%, with variability at 15.8% (moderate) and a slight increasing trend. Regarding the interchange subtest (M = 14), the stability was 80%, variability was at 9.8% (low), and the trend was increasing.

Participant E showed 85.7% stability, variability at 9.9% (low), and a slight increasing trend during the baseline phase (M = 11) of the dominant hand subtest. Regarding the non-dominant hand subtest (M = 20), the stability was 85.7% and variability was at 9.1% (low), with a slight decreasing trend. For the bimanual subtest (M = 5), the stability was 66.7%, with variability at 17.2% (low) and an increasing trend. Lastly, regarding the interchange subtest (M = 20), the stability was 85.7%, with variability at 9.1% (low) and a slight decreasing trend. During the intervention phase, the dominant hand subtest (M = 12) showed 80% stability, with variability at 7.9% (low) and an increasing trend. Regarding the non-dominant hand (M = 20), the stability was 80% and variability was at 8.6% (low), with a slight increasing trend. For the bimanual subtest (M = 5), the stability was 80%, with variability at 14.1% (low) and a slight increasing trend. Regarding the interchange subtest (M = 20), the stability was 80%, variability was at 8.6% (low), and the trend was flat.

#### 3.15.2. Between-Phase Visual Analysis

For Participant A, between-phase analysis showed an increase in level, an immediate effect, 0% overlap, moderate consistency, and a 100% PEM for the dominant hand subtest. Regarding the non-dominant hand subtest, there was a decrease in level, no clear immediacy of effect, 100% overlap, low consistency, and a 0% PEM. Regarding the bimanual subtest, there was no change in level, no immediacy of effect, 100% overlap, low consistency, and 0% PEM. Lastly, regarding the interchange subtest, there was an increase in level, no clear immediacy of effect, 0% overlap, moderate consistency, and a 100% PEM.

For Participant B, between-phase analysis showed no meaningful change in level, no clear immediacy of effect, 60% overlap, strong consistency, and a 40% PEM for the dominant hand subtest. Regarding the non-dominant hand subtest, there was no change in level, no clear immediacy of effect, 40% overlap, strong consistency, and a 60% PEM. For the bimanual subtest, there was an increase in level, an immediate effect, 0% overlap, moderate consistency, and a 100% PEM. Lastly, regarding the interchange subtest, there was a slight increase in level, no clear immediacy of effect, 20% overlap, strong consistency, and an 80% PEM.

For Participant C, between-phase analysis showed no change in level, no clear immediacy of effect, 60% overlap, low consistency, and a 40% PEM for the dominant hand subtest. Regarding the non-dominant hand subtest, there was no change in level, no clear immediacy of effect, 40% overlap, low consistency, and a 60% PEM. Regarding the bimanual subtest, there was no change in level, no clear immediacy of effect, 60% overlap, moderate consistency, and a 40% PEM. Lastly, regarding the interchange subtest, there was an increase in level, an immediate effect, 0% overlap, moderate consistency, and a 100% PEM.

For Participant D, between-phase analysis showed an increase in level, an immediate effect, 0% overlap, strong consistency, and a 100% PEM for the dominant hand subtest. Regarding the non-dominant hand subtest, there was an increase in level, no clear immediacy of effect, 20% overlap, strong consistency, and an 80% PEM. For the bimanual subtest, there was a slight increase in level, no clear immediacy of effect, 40% overlap, moderate consistency, and a 60% PEM. Lastly, regarding the interchange subtest, there was a slight increase in level, no clear immediacy of effect, 40% overlap, strong consistency, and a 60% PEM.

For Participant E, between-phase analysis showed a slight increase in level, no clear immediacy of effect, 20% overlap, strong consistency, and an 80% PEM for the dominant hand subtest. Regarding the non-dominant hand subtest, there was no change in level, no clear immediacy of effect, 40% overlap, strong consistency, and a 60% PEM. For the bimanual subtest, there was no change in level, no clear immediacy of effect, 40% overlap, strong consistency, and a 60% PEM. Lastly, regarding the interchange subtest, there was no change in level, no clear immediacy of effect, 60% overlap, strong consistency, and a 40% PEM.

### 3.16. Hand-Held Dynamometer

#### 3.16.1. Within-Phase Visual Analysis

For the left upper limb, Participant A showed 66.7% stability, variability at 20.8% (moderate), and an increasing trend during the baseline phase (M = 3.50) of the shoulder flexors subtest. Regarding the shoulder extensors subtest (M = 3.80), the stability was 66.7% and variability was at 15.6% (low), with an increasing trend. For the shoulder internal rotators subtest (M = 5.50), the stability was also 66.7%, with variability at 30.5% (moderate) and an increasing trend. Lastly, regarding the shoulder external rotators subtest (M = 4.10), the stability was 100%, with variability at 10.9% (low) and a slight increasing trend. During the intervention phase, the shoulder flexors subtest (M = 3.80) showed 100% stability, with variability at 8.5% (low) and a slight increasing trend. Regarding the shoulder extensors (M = 4.90), the stability was 60% and variability was at 14% (low), with a decreasing trend. Regarding the shoulder internal rotators subtest (M = 6.70), the stability was 60%, with variability at 14% (low) and an increasing trend. Regarding the shoulder external rotators subtest (M = 4.80), the stability was 100%, variability was at 7.7% (low), and the trend was flat. Analysis for the right upper limb showed 66.7% stability, variability of 23.8% (moderate), and an increasing trend during the baseline phase (M = 4.70) of the shoulder flexors subtest. Regarding the shoulder extensors subtest (M = 4.10), the stability was 100% and variability was at 3.6% (low), with a flat trend. For the shoulder internal rotators subtest (M = 5.30), the stability was 66.8%, with variability at 17.6% (low) and an increasing trend. Lastly, regarding the shoulder external rotators subtest (M = 4.90), the stability was 66.7%, with variability at 13.7% (low) and an increasing trend. During the intervention phase, the shoulder flexors subtest (M = 5.50) showed 60% stability, with variability at 17% (low) and an increasing trend. Regarding the shoulder extensors (M = 5.20), the stability was 40% and variability was at 20.6% (moderate), with an increasing trend. Regarding the shoulder internal rotators subtest (M = 8.10), the stability was 80%, with variability at 5.3% (low) and an increasing trend. For the shoulder external rotators subtest (M = 6.60), the stability was 80%, variability was at 10.6% (low), and the trend was increasing.

For the left upper limb, Participant B showed 0% stability, variability of 22% (moderate), and no clear trend during the baseline phase (M = 6.65) of the shoulder flexors subtest. Regarding the shoulder extensors subtest (M = 6.80), the stability was 75% and variability was at 13.3% (low), with a slight increasing trend. For the shoulder internal rotators subtest (M = 8.85), the stability was 75%, with variability at 8% (low) and an increasing trend. Lastly, regarding the shoulder external rotators subtest (M = 8.30), the stability was 75%, with variability at 12.9% (low) and an increasing trend. During the intervention phase, the shoulder flexors subtest (M = 9.80) showed 80% stability, with variability at 10.4% (low) and an increasing trend. Regarding the shoulder extensors (M = 8.40), the stability was 100% and variability was at 9.5% (low), with an increasing trend. For the shoulder internal rotators subtest (M = 11.20), the stability was 100%, with variability at 8.1% (low) and an increasing trend. Regarding the shoulder external rotators subtest (M = 10.50), the stability was 80%, variability was at 12.5% (low), and the trend was increasing. The right upper limb showed 100% stability, variability at 10.9% (low), and an increasing trend during the baseline phase (M = 6.90) of the shoulder flexors subtest. Regarding the shoulder extensors subtest (M = 8.45), the stability was 50% and variability was at 35.2% (moderate), with no clear trend. For the shoulder internal rotators subtest (M = 9.70), the stability was 100%, with variability at 3.4% (low) and a flat trend. Lastly, regarding the shoulder external rotators subtest (M = 8.60), the stability was 100%, with variability at 7.1% (low) and a slight increasing trend. During the intervention phase, the shoulder flexors subtest (M = 10.10) showed 100% stability, with variability at 7.7% (low) and an increasing trend. Regarding the shoulder extensors (M = 6.60), the stability was 80% and variability was at 10.8% (low), with a flat trend. For the shoulder internal rotators subtest (M = 10.60), the stability was 100%, with variability at 6.8% (low) and an increasing trend. Regarding the shoulder external rotators subtest (M = 10.20), the stability was 100%, variability was at 9.4% (low), and the trend was increasing.

For the left upper limb, Participant C showed 80% stability, variability at 17.7% (low), and a slight decreasing trend during the baseline phase (M = 12.50) of the shoulder flexors subtest. Regarding the shoulder extensors subtest (M = 9.90), the stability was 60% and variability was at 12.7% (low), with a slight increasing trend. For the shoulder internal rotators subtest (M = 12.90), the stability was 60%, with variability at 15.7% (low) and an increasing trend. Lastly, regarding the shoulder external rotators subtest (M = 10.10), the stability was 60%, with variability at 18.1% (low) and an increasing trend. During the intervention phase, the shoulder flexors subtest (M = 14.90) showed 100% stability, with variability at 3.9% (low) and an increasing trend. Regarding the shoulder extensors (M = 7.80), the stability was 100% and variability was at 6.2% (low), with an increasing trend. For the shoulder internal rotators subtest (M = 17.90), the stability was 100%, with variability at 11.3% (low) and an increasing trend. Regarding the shoulder external rotators subtest (M = 10.30), the stability was 80%, variability was at 11.6% (low), and the trend was increasing. Analysis for the right upper limb showed 60% stability, variability at 23.1% (moderate), and an increasing trend during the baseline phase (M = 15.80) of the shoulder flexors subtest. Regarding the shoulder extensors subtest (M = 12.10), the stability was 80% and variability was at 16.5% (low), with an increasing trend. For the shoulder internal rotators subtest (M = 13.90), the stability was 80%, with variability at 12.2% (low) and an increasing trend. Lastly, regarding the shoulder external rotators subtest (M = 13), the stability was 60%, with variability at 14.3% (low) and an increasing trend. During the intervention phase, the shoulder flexors subtest (M = 17.10) showed 100% stability, with variability at 7.3% (low) and an increasing trend. Regarding the shoulder extensors (M = 11), the stability was 80% and variability was at 11.7% (low), with no clear trend. For the shoulder internal rotators subtest (M = 18.30), the stability was 60%, with variability at 15.3% (low) and an increasing trend. Regarding the shoulder external rotators subtest (M = 12.10), the stability was 100%, variability was at 6.3% (low), and the trend was flat.

For the left upper limb, Participant D showed 33.3% stability, variability of 20.8% (moderate), and an increasing trend during the baseline phase (M = 7.55) of the shoulder flexors subtest. Regarding the shoulder extensors subtest (M = 8.60), the stability was 83.3% and variability was at 13.3% (low), with an increasing trend. For the shoulder internal rotators subtest (M = 14.55), the stability was 66.7%, with variability at 14.5% (low) and an increasing trend. Lastly, regarding the shoulder external rotators subtest (M = 10.75), the stability was 83.3%, with variability at 11.7% (low) and an increasing trend. During the intervention phase, the shoulder flexors subtest (M = 8.70) showed 100% stability, with variability at 5.2% (low) and an increasing trend. For the shoulder extensors (M = 11.50), the stability was 80% and variability was at 12.9% (low), with an increasing trend. Regarding the shoulder internal rotators subtest (M = 14.50), the stability was 100%, with variability at 7.7% (low) and a flat trend. Regarding the shoulder external rotators subtest (M = 12.30), the stability was 100%, variability was at 7.4% (low), and the trend was increasing. The right upper-limb analysis showed 50% stability, variability at 23% (moderate), and an increasing trend during the baseline phase (M = 6.80) of the shoulder flexors subtest. Regarding the shoulder extensors subtest (M = 5.25), the stability was 66.8% and variability was at 17.3% (low), with an increasing trend. Regarding the shoulder internal rotators subtest (M = 9.30), the stability was 50%, with variability at 26.5% (moderate) and an increasing trend. Lastly, regarding the shoulder external rotators subtest (M = 7.35), the stability was 66.7%, with variability at 14.7% (low) and an increasing trend. During the intervention phase, the shoulder flexors subtest (M = 9.90) showed 60% stability, with variability at 14.6% (low) and an increasing trend. Regarding the shoulder extensors (M = 6.30), the stability was 60% and variability was at 20% (low), with an increasing trend. Regarding the shoulder internal rotators subtest (M = 12.60), the stability was 100%, with variability at 4.4% (low) and an increasing trend. For the shoulder external rotators subtest (M = 9.20), the stability was 60%, variability was at 13.9% (low), and the trend was increasing.

For the left upper limb, Participant E showed 85.7% stability, variability at 15.2% (low), and a slight increasing trend during the baseline phase (M = 13.50) of the shoulder flexors subtest. Regarding the shoulder extensors subtest (M = 12.60), the stability was 85.7%, and variability was at 7.9% (low), with a slight increasing trend. For the shoulder internal rotators subtest (M = 13.30), the stability was 100%, with variability at 4.3% (low) and a flat trend. Lastly, regarding the shoulder external rotators subtest (M = 12.60), the stability was 85.71%, with variability at 7.9% (low) and a slight increasing trend. During the intervention phase, the shoulder flexors subtest (M = 15) showed 100% stability, with variability at 8.4% (low) and an increasing trend. For the shoulder extensors (M = 14.10), the stability was 100%, and variability was at 4.1% (low), with an increasing trend. Regarding the shoulder internal rotators subtest (M = 15.10), the stability was 100% with variability at 2.2% (low) and an increasing trend. Regarding the shoulder external rotators subtest (M = 14.10), the stability was 100%, variability was at 4.1% (low), and the trend was increasing. In the right upper limb, analysis showed 71.4% stability, variability of 14.6% (low), and a slight increasing trend during baseline phase (M = 13) of the shoulder flexors subtest. Regarding the shoulder extensors subtest (M = 12.50), the stability was 57.1%, and variability was at 15.9% (low), with an increasing trend. For the shoulder internal rotators subtest (M = 13.10), the stability was 85.7%, with variability at 7.7% (low) and a flat to slightly increasing trend. Lastly, regarding the shoulder external rotators subtest (M = 12.50), the stability was 57.1%, with variability at 15.9% (low) and an increasing trend. During the intervention phase, the shoulder flexors subtest (M = 13.50) showed 80% stability, with variability at 9.9% (low) and an increasing trend. Regarding the shoulder extensors (M = 12.60), the stability was 60%, and variability was at 17.2% (low), with a slight increasing trend. For the shoulder internal rotators subtest (M = 14.80), the stability was 100%, with variability at 4.6% (low) and an increasing trend. Regarding the shoulder external rotators subtest (M = 12.60), the stability was 60%, variability was at 17.2% (low), and the trend was slightly increasing.

#### 3.16.2. Between-Phase Visual Analysis

For Participant A’s left upper limb, between-phase analysis showed an increase in level, no clear immediacy of effect, 20% overlap, strong consistency, and an 80% PEM for the shoulder flexors subtest. Regarding the shoulder extensors subtest, there was an increase in level, an immediate effect, 0% overlap, moderate consistency, and a 100% PEM. Regarding the shoulder internal rotators subtest, there was an increase in level, no clear immediacy of effect, 20% overlap, moderate consistency, and an 80% PEM. Lastly, regarding the shoulder external rotators subtest, there was an increase in level, an immediate effect, 0% overlap, strong consistency, and a 100% PEM. For Participant A’s right upper limb, between-phase analysis showed an increase in level, no clear immediacy of effect, 20% overlap, moderate consistency, and an 80% PEM for the shoulder flexors subtest. Regarding the shoulder extensors subtest, there was an increase in level, an immediate effect, 0% overlap, moderate consistency, and a 100% PEM. For the shoulder internal rotators subtest, there was an increase in level, an immediate effect, 0% overlap, strong consistency, and a 100% PEM. Lastly, regarding the shoulder external rotators subtest, there was an increase in level, an immediate effect, 0% overlap, strong consistency, and a 100% PEM.

For Participant B’s left upper limb, between-phase analysis showed an increase in level, an immediate effect, 0% overlap, moderate consistency, and a 100% PEM for the shoulder flexors subtest. Regarding the shoulder extensors subtest, there was an increase in level, an immediate effect, 0% overlap, strong consistency, and a 100% PEM. For the shoulder internal rotators subtest, there was an increase in level, an immediate effect, 0% overlap, strong consistency, and a 100% PEM. Lastly, regarding the shoulder external rotators subtest, there was an increase in level, an immediate effect, 0% overlap, strong consistency, and a 100% PEM. For Participant B’s right upper limb, between-phase analysis showed an increase in level, an immediate effect, 0% overlap, strong consistency, and a 100% PEM for the shoulder flexors subtest. Regarding the shoulder extensors subtest, there was a decrease in level, no clear immediacy of effect, 100% overlap, moderate consistency, and a 0% PEM. For the shoulder internal rotators subtest, there was an increase in level, no clear immediacy of effect, 20% overlap, strong consistency, and a 80% PEM. Lastly, regarding the shoulder external rotators subtest, there was an increase in level, an immediate effect, 0% overlap, strong consistency, and a 100% PEM.

For Participant C’s left upper limb, between-phase analysis showed an increase in level, an immediate effect, 0% overlap, strong consistency, and a 100% PEM for the shoulder flexors subtest. Regarding the shoulder extensors subtest, there was a decrease in level, no clear immediacy of effect, 100% overlap, strong consistency, and a 0% PEM. For the shoulder internal rotators subtest, there was an increase in level, an immediate effect, 0% overlap, strong consistency, and a 100% PEM. Lastly, regarding the shoulder external rotators subtest, there was a slight increase in level, no clear immediacy of effect, 40% overlap, strong consistency, and a 60% PEM. For Participant C’s right upper limb, between-phase analysis showed an increase in level, no clear immediacy of effect, 20% overlap, strong consistency, and an 80% PEM for the shoulder flexors subtest. Regarding the shoulder extensors subtest, there was a decrease in level, no clear immediacy of effect, 80% overlap, strong consistency, and a 20% PEM. For the shoulder internal rotators subtest, there was an increase in level, an immediate effect, 0% overlap, strong consistency, and a 100% PEM. Lastly, regarding the shoulder external rotators subtest, there was a decrease in level, no clear immediacy of effect, 100% overlap, strong consistency, and a 0% PEM.

For Participant D’s left upper limb, between-phase analysis showed an increase in level, an immediate effect, 0% overlap, moderate consistency, and a 100% PEM for the shoulder flexors subtest. Regarding the shoulder extensors subtest, there was an increase in level, an immediate effect, 0% overlap, strong consistency, and a 100% PEM. For the shoulder internal rotators subtest, there was no meaningful change in level, no clear immediacy of effect, 60% overlap, strong consistency, and a 40% PEM. Lastly, regarding the shoulder external rotators subtest, there was an increase in level, an immediate effect, 20% overlap, strong consistency, and an 80% PEM. For Participant D’s right upper limb, between-phase analysis showed an increase in level, an immediate effect, 0% overlap, moderate consistency, and a 100% PEM for the shoulder flexors subtest. Regarding the shoulder extensors subtest, there was an increase in level, an immediate effect, 0% overlap, moderate consistency, and a 100% PEM. For the shoulder internal rotators subtest, there was an increase in level, an immediate effect, 0% overlap, strong consistency, and a 100% PEM. Lastly, regarding the shoulder external rotators subtest, there was an increase in level, an immediate effect, 0% overlap, strong consistency, and a 100% PEM.

For Participant E’s left upper limb, between-phase analysis showed an increase in level, no clear immediacy of effect, 20% overlap, strong consistency, and an 80% PEM for the shoulder flexors subtest. Regarding the shoulder extensors subtest, there was an increase in level, an immediate effect, 0% overlap, strong consistency, and a 100% PEM. For the shoulder internal rotators subtest, there was an increase in level, an immediate effect, 0% overlap, strong consistency, and a 100% PEM. Lastly, regarding the shoulder external rotators subtest, there was an increase in level, an immediate effect, 0% overlap, strong consistency, and a 100% PEM. For Participant E’s right upper limb, between-phase analysis showed a slight increase in level, no clear immediacy of effect, 40% overlap, strong consistency, and a 60% PEM for the shoulder flexors subtest. Regarding the shoulder extensors subtest, there was no meaningful change in level, no clear immediacy of effect, 40% overlap, strong consistency, and a 60% PEM. Regarding the shoulder internal rotators subtest, there was an increase in level, an immediate effect, 0% overlap, strong consistency, and a 100% PEM. Lastly, regarding the shoulder external rotators subtest, there was no meaningful change in level, no clear immediacy of effect, 40% overlap, strong consistency, and a 60% PEM.

### 3.17. Symbol Digit Modalities Test

#### 3.17.1. Within-Phase Visual Analysis

Participant A showed 100% stability across phases (baseline, M = 35; intervention, M = 42), with baseline variability at 18.7% (low), showing an upward trend, and intervention variability at 1.9% (low), with a stable trend. Participant B also demonstrated 100% stability (baseline, M =47; intervention, M = 51.8), with baseline variability at 6.4% (low), with an upward trend, and intervention variability at 1.7% (low), with a stable trend. Participant C had 100% stability across phases (baseline; M = 27, intervention; M = 32), with baseline variability of 4% (low) and upward trend and intervention variability of 2.8% (low) and a stable trend. Participant D maintained 100% stability across phases (baseline; M = 33, intervention; M = 36), with baseline variability of 16.5% (low) and stable trend and intervention variability of 3.1% (low) and stable trend. Participant E showed 99.9% stability across phases (baseline; M = 28, intervention; M = 28) with baseline variability of 4.3% (low) and downward trend and intervention variability of 9.3% (low) and upward trend.

#### 3.17.2. Between Phase Visual Analysis

Participant A demonstrated PEM of 100%, no immediacy, moderate consistency, and a level change of 4.2 points with 80% overlap. Participant B achieved PEM of 100% with immediate effect, consistent performance, a level increase of 4.67 points with 0% overlap. Participant C showed PEM of 100% high immediacy, high consistency, and a level increase of 5 points, and no overlap. Participant D achieved PEM of 80%, strong immediate effect, upward consistency, a level increase of 2 points with 20% overlap. Participant E showed PEM of 100%, immediate effect, moderate consistency, and a level shift of 5 points with no overlap.

### 3.18. Pinch Test

#### 3.18.1. Within Phase Visual Analysis

Participant A demonstrated 100% stability in both upper limbs at baseline (left; M = 1.5, right; M = 2.1) with 69.5% (high) variability and a downward trend in the left upper limb. The right limb had a variability of 5.8% (low) and a stable trend. During intervention (left; M = 2, right; M = 3.1), stability for both upper limbs was 80% with variability of 33.8% (moderate) and an upward trend for the left upper limb and 28.1% (moderate) and an upward trend for the right upper limb. Participant B showed 75% (right baseline; M = 12, intervention; M = 13) stability for both upper limbs, with 31% (moderate) variability and a downward trend in the left (baseline; M = 8, intervention; M = 9) upper limb and 19.2% (low) variability and an upward trend for the right upper limb. Participant C showed a baseline (M = 12.5), stability of 20% with 56.8% (high) variability, and an upward trend in the left upper limb. In the right upper limb baseline (M = 14.3), the stability was 60% with 46.6% (high) variability and upward trend. Intervention (left; M = 15.7, right; M = 15.2) stability was 100% in both limbs, with variability 9.4% (low) (left) and 14.6% (low) (right), both showing upward trends. Participant D presented baseline stability 50%, variability 32.5% (moderate), and an upward trend for the left upper limb (M = 11.3) and 66.7% stability, 39.5% (moderate) variability, and an upward trend for the right upper limb (M = 10). During intervention, stability reached 100% bilaterally (left; M = 15.3, right; M = 13.7), and the trend was stable bilaterally, while intervention was 8.3% (low) (left) and 5.4% (low) (right). Participant E had baseline stability of 99.6% for both upper limbs (left; M = 11.8, right; M = 17) across both phases, variability 28.88% (moderate), and a stable trend for the left upper limb and 37.3% (moderate) variability and upward trend for the right. Intervention (left; M = 17.3, right; M = 20.3) variability was 9.71% (low) with upward trend (left) and 19.8% (low) with upward trend (right).

#### 3.18.2. Between Phase Visual Analysis

Participant A showed a left hand PEM of 60% with immediate improvement, upward consistency, and a level increase of 0.52 lbs with 60% overlap, while the right hand showed a PEM of 80%, immediate effect, upward consistency, and a level increase of 1.13, lbs with no overlap. Participant B demonstrated a PEM of 80% bilaterally, with no immediate effect, moderate consistency, a level increase of 0.43 lbs, and an overlap of 40% for both sides. Participant C showed a left hand PEM of 100%, with high immediacy of effect, upward consistency, and a level change of 1.93 lbs, with no overlap. The right hand had a PEM of 80%, moderate immediacy of effect, low consistency, and a level change of 0.77 lbs, with no overlap. Participant D achieved, across both hands, a PEM of 100%, with strong immediate upward shifts, consistent improvement, a level change of 0.96 lbs, and overlap of 20% for the left hand and 30% for the right. Participant E showed a left hand PEM of 100%, with strong immediacy, consistent improvement, and a level change of 3.9 lbs, with no overlap, while the right hand showed a PEM of 80%, immediate effect, lower consistency, and a level change of 0.3 lbs, with full overlap.

### 3.19. Questionnaires

Analysis of the MFIS scores revealed notable variability in individual responses to the intervention. Participant A showed a marked reduction in fatigue, with scores decreasing from 51 to 30 from baseline to intervention. Participant B’s fatigue levels remained unchanged, suggesting no measurable improvement. Participant C exhibited a minimal change, with scores dropping slightly from 4 to 1, indicating an already low fatigue level. Participant D demonstrated a moderate improvement, decreasing from 58 to 49, while Participant E experienced a substantial reduction from 32 to 2, representing the most pronounced improvement in the group.

Regarding the SF-36 quality of life questionnaire, the changes were less pronounced. Participant A’s score decreased marginally from 88 to 87, and Participant B’s from 98 to 96. Participant C’s score declined from 100 to 93, while Participant D’s score improved slightly from 82 to 88. Participant E experienced a small decline from 85 to 83.

### 3.20. Result of Statistical Analysis

Consistent with the analysis plan, only participants whose data fulfilled the visual assessment criteria were retained for statistical analysis. Based on the results of the five participants for the aMT, statistically significant differences were observed only in Participant A’s right APB, Participant D’s left APB, and Participant E’s left APB. The non-overlap of the analysis of all pairs indicated moderate, statistically significant improvements for Participant A’s right APB (NAP = 69.6%, *p* = 0.041). In contrast, Participant D’s left APB (NAP = 0%, *p* = 0.008) and Participant E’s left APB (NAP = 0%, *p* = 0.013) demonstrated complete non-overlap, with all intervention values lower than baseline values. Because lower aMT values indicate greater corticospinal excitability, these findings represent consistent improvements following the intervention.

Statistically significant changes were observed in the hand-held dynamometer assessment in Participant C’s left upper limb (shoulder flexors: NAP = 92%, *p* = 0.003; shoulder extensors: NAP = 6%, *p* = 0.003; shoulder internal rotators: NAP = 92%, *p* = 0.003) and right upper limb (shoulder internal rotators: NAP = 92%, *p* = 0.003). Significant changes were also identified in the SDMT in Participant C (NAP = 100%, *p* = 0.01) and Participant D (NAP = 86.7%, *p* = 0.02). Furthermore, statistically significant improvements were observed for the Mini-BEST test in Participant D (NAP = 84%, *p* = 0.03). In addition, the pinch test demonstrated significant changes in Participant D, in both the left upper limb (NAP = 96.7%, *p* = 0.01) and the right upper limb (NAP = 100%, *p* = 0.008).

## 4. Discussion

This pilot concurrent multiple-baseline study examined the effects of an in-phase bilateral upper-limb exercise protocol on corticospinal plasticity and clinical outcomes in pwPMS. Unlike RRMS, PMS is marked by a gradual accumulation of disability and reduced responsiveness to pharmacological therapies, with limited neuroplastic reserve [57]. Thus, neurorehabilitative strategies targeting motor and cognitive impairments are increasingly needed. The present findings provide preliminary individual-level evidence of functional change, integrating repeated TMS and behavioural measures within a rigorous single-case design. Given the exploratory nature of this pilot concurrent multiple-baseline single-case experimental design, the findings should be interpreted as preliminary and hypothesis-generating, providing a foundation for future controlled investigations.

### 4.1. The Effects of In-Phase Bilateral Exercises on Corticospinal Excitability

Changes in aMT suggested that in-phase bilateral upper-limb training may enhance corticospinal excitability in pwPMS. Although not all effects reached statistical significance, 4 out of 5 participants (except Participant B) showed a decreased aMT in one or both APBs, suggesting increased excitability in the corticospinal neurons and intracortical facilitatory circuits [58]. Such modulation may reflect the synchronous activation of homologous muscles during in-phase movements, which engage both hemispheres and promote the symmetrical excitatory drive across cortical and subcortical regions [59,60].

The observed interindividual variability may reflect differences in lesion burden, disease duration, or compensatory cortical reorganization [61,62]. An asymmetric aMT between limbs likely corresponds to uneven lesion distribution or habitual motor dominance [27,63]. Previous studies suggest that in-phase bilateral training may reduce inhibitory interhemispheric demands and activate supplementary and premotor cortical regions that project bilaterally to spinal motor neurons, thereby potentially supporting excitability in lesioned areas [28,29]. Previous studies have also reported that repetitive task-specific activity increases expression of brain-derived neurotropic factor and insulin-like growth factor-1 [64,65], both of which lower motor thresholds by enhancing excitatory transmission and axonal growth while reducing GABAergic inhibition [66,67]. These mechanisms may represent one possible explanation for the neurophysiological changes observed at the end of the intervention [24,25]. Further studies involving larger cohorts and direct neurochemical assessments are required to clarify the neurobiological mechanisms underlying the observed changes in corticospinal excitability.

Variability across participants likely reflects both neurophysiological and methodological constraints. In PMS, demyelination and axonal loss limit the number of responsive corticospinal projections, constraining excitability modulation [68,69]. Small deviations in coil placement and fatigue-related fluctuations in voluntary contraction could also obscure subtle changes. These considerations are important for interpreting TMS-derived measures in this population. Additionally, the variability in treatment response observed across participants may partly reflect differences in baseline clinical characteristics, including age, disability level, and disease duration, in addition to individual differences in responsiveness to the intervention.

Compared with our previous work [24], which reported stronger and more immediate rMT reductions in RRMS, the current PMS cohort exhibited smaller, slower, and more variable responses, which may be consistent with reduced plastic reserve due to chronic demyelination and axonal loss [14,68,69]. Nevertheless, both studies reported functional improvements, suggesting that bilateral motor interventions may be feasible across different MS phenotypes, although confirmation in larger controlled studies is required. Similarly, Chaves et al. (2020) [25] reported greater aMT reductions after aerobic treadmill exercise, likely due to broader sensorimotor network engagement, which were observed mostly in the dominant hemisphere. Future comparisons across exercise modalities may clarify optimal approaches for enhancing corticospinal excitability in PMS.

The variability in responses observed across participants highlights the heterogeneous nature of PMS [14] and its influence on rehabilitation outcomes. Differences in age, disease duration, disability level, and underlying neural integrity may have contributed to the differential responsiveness to the intervention [14]. In particular, the extent of residual corticospinal tract integrity likely plays a critical role in determining the capacity for neurological adaptation [10,14,69]. Participants with a greater preserved neural reserve may be more likely to exhibit measurable changes in corticospinal excitability, whereas others may rely more heavily on compensatory mechanisms to achieve functional improvements. Additionally, asymmetrical findings between limbs further support the influence of lesion distribution and habitual motor dominance on training response [27,63]. Rather than being solely a limitation, this variability underscores the need for individualised rehabilitation approaches in PMS, where interventions may need to be tailored based on patient-specific characteristics.

Although follow-up assessments were not included in the present study, previous work using the same intervention [24] demonstrated that improvements in motor threshold were not maintained after cessation of the programme, suggesting that continued exercise may be required to sustain neurophysiological adaptations. Thus, follow-up assessments were beyond the scope of the present proof-of-concept study.

### 4.2. Effects on Clinical Outcomes

Bilateral training was associated with measurable functional improvements, most consistently observed in muscle strength, indicating enhanced motor unit recruitment and neuromuscular coordination. Gains in SDMT performance suggest improvements in cognitive processing speed, which may be related to enhanced interhemispheric communication or engagement of attentional networks, as proposed in previous studies [70,71]. These findings are consistent with the possibility of cross-domain effects of motor training, potentially mediated by shared neural substrates involved in motor and cognitive function [71]. Even when TMS indices showed modest changes, behavioural improvements may reflect compensatory recruitment of parallel motor pathways or enhanced efficiency within preserved neural circuits, although these mechanisms were not directly assessed in the present study [72,73].

Importantly, these changes are likely to be clinically meaningful, as improvements in upper-limb strength and fine motor control are directly associated with the ability to perform activities of daily living, including grasping, manipulation, and object handling. The predominance of gains in these domains likely reflects the principle of training specificity, given that the intervention primarily involved repetitive, task-oriented bilateral upper-limb movements. In addition to motor improvements, the observed enhancement in SDMT performance suggests that exercise may exert broader effects on cognitive processing, potentially through increased interhemispheric communication and engagement of shared motor–cognitive networks.

Where established MS-specific benchmarks were available, the magnitude of change was also considered, alongside statistical significance. For the SDMT, a change of approximately 4 raw-score points, or 10%, has been proposed as clinically meaningful in people with multiple sclerosis [51]. Participant C exceeded both of these thresholds, whereas the statistically significant improvement observed in Participant D did not clearly reach these benchmarks. Nevertheless, these findings should be interpreted cautiously, given the pilot nature of the study, the small sample size, and the potential influence of repeated testing. No established minimal clinically important differences (MCIDs) currently exist for a TMS-derived active motor threshold or for the hand-held dynamometry measures used in the present study. Consequently, changes in these outcomes should be interpreted as preliminary neurophysiological and functional improvements rather than definitive evidence of clinically meaningful benefit.

Although the present study was not powered to establish clinically important differences, the observed improvements in grip strength, manual dexterity, balance, fatigue, and cognitive processing speed may nevertheless be clinically meaningful. Even modest gains in upper-limb strength and dexterity can facilitate everyday activities such as grasping, carrying objects, self-care, and household tasks, while improvements in balance and fatigue may contribute to safer mobility and greater participation in daily life. Similarly, enhanced cognitive processing speed may positively influence attention and functional independence. Given the progressive nature of PMS, where maintaining functional independence is often considered a primary rehabilitation goal, even modest functional improvements or stabilization across multiple domains may represent meaningful therapeutic benefits.

However, improvements were not uniform across all clinical measures, which may be attributed to differences in baseline participant characteristics (age, disease duration, etc.) as mentioned previously, as well as the specific neural systems targeted by the intervention. Notably, even the modest functional gains are of particular importance in pwPMS, where disability typically accumulates over time, and opportunities for recovery are limited.

It is important to mention that to analyse the data from the hand-held dynamometry test, two pairs of agonist–antagonist muscles were used. This was done because the hand-held dynamometry assessment provides an estimate of net joint torque generated during a specific movement rather than the force of individual muscles [74]. As such, the recorded force reflects the combined contribution of agonist muscles and the opposing influence of antagonist muscles, acting across the joint [75]. In line with established biomechanical principles, joint torque production is determined by the interaction between opposing muscle groups [75]. This approach is consistent with prior upper-limb strength research, where muscle performance is commonly evaluated at the level of opposing movement groups rather than for individual muscles [74].

The principal objective of this pilot proof-of-concept study was to investigate the neurophysiological effects of the intervention; therefore, corticospinal excitability was selected as the primary outcome. Nevertheless, the accompanying improvements observed in patient-centred outcomes provide important insight into the intervention’s potential clinical relevance. Consistent with the methodology of our previous study investigating the same intervention [24], clinical outcomes were included as secondary measures to explore whether measurable neurophysiological adaptations were accompanied by improvements in upper-limb performance, fatigue, cognitive processing speed, balance, and quality of life. While the present study was not designed to establish causal relationships between neurophysiological and functional changes, the concurrent improvements observed across these domains support further investigation of this intervention in larger controlled studies specifically designed to examine these associations.

It is important to note that improvements in neurophysiological and clinical outcomes were not consistently observed in the same participants. Therefore, although beneficial changes were identified across both domains, the present study was not designed to determine whether changes in corticospinal excitability directly mediated the observed functional improvements. Future studies with larger samples are warranted to investigate the relationship between neurophysiological adaptations and clinical recovery following in-phase bilateral exercise.

### 4.3. Feasibility and Tolerability of the Intervention

Beyond efficacy, the present study provides important evidence regarding the feasibility and tolerability of a structured in-phase bilateral exercise program in pwPMS. All participants completed the 12-week intervention period without adverse events, indicating that the exercise protocol was safe and well-tolerated, even in individuals with moderate disability levels. This is particularly relevant in the context of PMS, where fatigue, mobility limitations, and disease burden often restrict participation in sustained exercise programs. The ability of participants to adhere to a relatively high-volume protocol supports the practicality of incorporating similar interventions into clinical rehabilitation settings. These findings also suggest that appropriately tailored exercise programs can be safely implemented in pwPMS and may serve as a valuable adjunct to standard care. Similar outcomes regarding the feasibility of the exercise programme were observed in our previous work, in which all participants were able to complete the 12-week in-phase bilateral upper-limb exercise programme [24]. Therefore, the present exercise protocol not only demonstrated beneficial effects but also reinforces the feasibility of delivering consistent, structured exercise to this clinical population.

From a clinical perspective, the present intervention should be considered an adjunct rather than an alternative to disease-modifying therapies [76]. While pharmacological treatments primarily aim to reduce inflammatory activity and slow disease progression, rehabilitation interventions target functional impairments, activity limitations, participation, and quality of life [76]. This distinction may be particularly relevant for people with progressive multiple sclerosis, in whom therapeutic options remain relatively limited, and rehabilitation plays a central role in maintaining functional independence [77]. Consequently, in-phase bilateral upper-limb exercise may represent a practical complementary strategy within multidisciplinary MS management rather than a replacement for established medical therapy [78].

### 4.4. Limitations

Several methodological considerations should be acknowledged. First, TMS devices have a maximum output of 78% MSO, which limited the assessment of very high motor thresholds, possibly leading to an underestimation in participants with reduced corticospinal excitability. However, this limitation did not affect the present results (mean aMT (MSO %): left, 25.56; right, 25.60), as none of the aMT measurements reached the specific threshold. Furthermore, although the active motor threshold was selected as the primary neurophysiological outcome, based on previous work demonstrating intervention-related changes in resting motor threshold following the same bilateral in-phase exercise programme [24], it reflects only one aspect of corticospinal excitability. Future studies should incorporate additional TMS measures, such as short-interval intracortical inhibition, intracortical facilitation, a cortical silent period, and recruitment curves, to provide a more comprehensive characterization of cortical plasticity. Second, the absence of a neuronavigation system may have introduced variability in coil placement; however, all sessions were conducted by the same trained assessor using standardized scalp measurements to minimize this variability and ensure consistency in stimulation delivery and cortical excitability targeting [78]. Third, the small sample size, together with clinical heterogeneity regarding age, disability level (EDSS), and disease duration, as well as the absence of an external control group, limits the generalizability of the findings. Although the multiple-baseline single-case experimental design strengthens internal validity by allowing each participant to serve as their own control through repeated baseline and intervention measurements, the absence of an external control group reduces the strength of causal inference. Importantly, even though visual analysis is a cornerstone of single-case experimental design methodology, selecting datasets for subsequent statistical analysis following visual inspection may introduce a degree of confirmation bias; therefore, visual findings were complemented by objective quantitative analyses (NAP and randomization tests). Finally, although the Bonferroni correction was applied to control for multiple statistical comparisons and reduce the risk of Type I errors, its conservative nature may have increased the likelihood of Type II errors, particularly given the exploratory nature of this pilot study and the small sample size. Therefore, these findings should be considered preliminary and require replication in larger, controlled studies. Nevertheless, the present design is appropriate for proof-of-concept investigations and is consistent with established methodological recommendations for single-case experimental research [31,32,33].

### 4.5. Recommendation for Future Research

(1) Future studies should incorporate neuronavigation systems to improve precision in hotspot localization and use stimulators capable of reaching higher output ranges. (2) Recruitment criteria should aim for greater homogeneity regarding age, sex, and disease duration to reduce intersubject variability, and larger cohorts are needed to confirm generalizability. (3) Randomized controlled designs comparing different exercise modalities, i.e., previously published types of exercises versus this specific in-phase bilateral upper-limb exercise protocol, under matched conditions, would clarify which type best enhances corticospinal excitability in pwPMS. (4) Including a control group and follow-ups could further establish causality and the durability of the neuroplastic and functional effects.

## 5. Conclusions

This pilot study provides preliminary evidence that in-phase bilateral upper-limb exercises may modulate corticospinal excitability and improve functional performance in some pwPMS. Despite interindividual variability, a general trend toward lower aMT values was observed across participants, which may reflect enhanced cortical excitability and residual plastic potential in this clinical cohort. Observed improvements in strength and cognitive processing further suggest that in-phase bilateral, task-specific training may contribute to cross-domain functional gains, even in advanced disease stages.

These findings highlight the potential of non-pharmacological, exercise-based interventions to complement existing neurorehabilitation approaches in PMS. While limited by a small sample size, the study underscores the feasibility and clinical relevance of using single-case experimental designs to explore neuroplastic mechanisms in progressive neurological conditions. Larger, controlled clinical trials can be used to validate these effects, define optimal exercise parameters, and determine the long-term impact of such exercises on neural and behavioural outcomes.

## Figures and Tables

**Table 1:** In-phase exercise program: The protocol was delivered in a circuit format consisting of three consecutive cycles per session. Each exercise was performed for a minimum of 20 repetitions, with exercise intensity monitored via heart rate and maintained below 75% of age-predicted maximum heart rate. Progression was individualized across sessions through adjustments to resistance, repetitions, or throwing distance, while preserving the in-phase bilateral execution of upper-limb exercises. Depending on the abilities of the participants, the exercises were performed either standing or seated. ABD = abduction; ER = external rotation; IR = internal rotation; ADD = adduction; F = flexion.

In-Phase Bilateral Exercise Program		
Exercise	Repetitions	Level of Difficulty
Basketball chest pass	≥20 repetitions	Distance/number of repetitions
PNF 1st diagonal flexion pattern	≥20 repetitions	Resistance/number of repetitions
Finger flexion using resistance net	≥20 repetitions	Resistance/number of repetitions
ADD + IR of legs (seated position/hips and knees in F/Pilates ring)	≥20 repetitions (per cycle)	Number of repetitions
Basketball shoulder pass	≥20 repetitions (per cycle)	Distance/number of repetitions
PNF 1st diagonal extension pattern	≥20 repetitions (per cycle)	Resistance/number of repetitions
Finger extension using resistance net	≥20 repetitions (per cycle)	Resistance/number of repetitions
ABD + ER of legs (seated position/hips and knees in F/Pilates ring)	≥20 repetitions (per cycle)	Number of repetitions
Basketball chest pass	≥20 repetitions (per cycle)	Distance/number of repetitions
PNF 2nd diagonal flexion pattern	≥20 repetitions (per cycle)	Resistance/number of repetitions
PNF 2nd diagonal extension pattern	≥20 repetitions (per cycle)	Resistance/number of repetitions
Squat	≥20 repetitions (per cycle)	Number of repetitions

**Table 2:** Demographic characteristics of the participants: The age of the participants ranged from 46 to 74, with fluctuations in height and weight. Upper limbs, as well as the HR, were measured using the same method for all participants. EDSS scores were similar for participants A to D, with participant E having a lower EDSS score. Participants A, B, C and E engaged in sedentary work, while Participant D engaged in accounting. BMI: body mass index; HR: heart rate; EDSS: Expanded Disability Status Scale; MS: multiple sclerosis.

Demographic Characteristics of the Participants					
Participant	A	B	C	D	E
**Age (years)**	74	60	53	46	72
**Sex**	Female	Female	Male	Male	Male
**Dominant Hand**	Left	Right	Right	Right	Right
**Weight (kg)**	53	63	100	88	79
**Height (m)**	1.59	1.53	1.76	1.80	1.54
**BMI**	21 (normal)	26.9 (overweight)	32.3 (overweight)	27.2 (overweight)	33.3 (overweight)
**HRmax (bpm)**	146	160	167	174	148
**Left Upper-limb Length (cm)**	85	74	102	100	97
**Right Upper-limb Length** **(cm)**	83	75	103	102	96
**EDSS**	6.5	6	6	6	3
**Disease Duration**	28 years	23 years	10 years	18 years	43 years
**Current Clinical Symptoms**	- Upper- and lower-limb weakness - Spasticity	- Lower-limb weakness - Imbalance during gait - Spasticity	- Lower-limb weakness - Imbalance during gait - Spasticity	- Lower-limb weakness - Imbalance during gait - Spasticity	- Mild lower-limb weakness
**MS-Related Medication**	Baclofen	Rituximab, Fampyra	Fampyra	Rituximab, Fampyra	Interferone
**Occupation**	Sedentary	Sedentary	Sedentary	Accountant	Sedentary

## Data Availability

The data presented in this study are openly available at (https://doi.org/10.6084/m9.figshare.32098522).

## Appendix A

**Table A1:** Active motor threshold raw data: the data are shown for each assessment point during both the baseline and intervention phase. The numbers represent the percentage of amplitude of the MSO. N/A: not available; MSO: maximum stimulator output; APB: abductor pollicis brevis.

Participant & APB	A-Left	A-Right	B-Left	B-Right	C-Left	C-Right	D-Left	D-Right	E-Left	E-Right
**Baseline 1**	23	48	18	18	48	36	35	34	18	19
**Baseline 2**	19	31	26	21	30	20	24	39	41	21
**Baseline 3**	23	41	18	19	47	23	25	52	18	20
**Baseline 4**	N/A	N/A	18	21	32	20	27	28	19	18
**Baseline 5**	N/A	N/A	N/A	N/A	42	24	37	26	18	39
**Baseline 6**	N/A	N/A	N/A	N/A	N/A	N/A	38	39	34	18
**Baseline 7**	N/A	N/A	N/A	N/A	N/A	N/A	N/A	N/A	19	18
**Intervention 1**	19	34	19	32	36	23	18	36	17	17
**Intervention 2**	18	20	26	19	45	28	21	26	44	18
**Intervention 3**	19	30	17	31	25	18	23	40	17	17
**Intervention 4**	22	25	18	22	31	17	18	22	17	17
**Intervention 5**	35	19	19	19	23	18	17	22	17	17
